# *bfb*, a Novel ENU-Induced *blebs* Mutant Resulting from a Missense Mutation in *Fras1*

**DOI:** 10.1371/journal.pone.0076342

**Published:** 2013-10-15

**Authors:** Kerry A. Miller, Christopher T. Gordon, Megan F. Welfare, Georgina Caruana, John F. Bertram, John F. Bateman, Peter G. Farlie

**Affiliations:** 1 Murdoch Children's Research Institute, Royal Children's Hospital, Parkville, Victoria, Australia; 2 Departments of Paediatrics, Biochemistry and Molecular Biology, University of Melbourne, Parkville, Victoria, Australia; 3 Department of Anatomy and Developmental Biology, Monash University, Clayton, Victoria, Australia; Pennington Biomedical Research Center/LSU, United States of America

## Abstract

Fras1 is an extracellular matrix associated protein with essential roles in adhesion of epithelia and mesenchyme during early embryonic development. The adhesive function of Fras1 is achieved through interaction with a group of related proteins, Frem 1–3, and a cytoplasmic adaptor protein Grip1. Mutation of each of these proteins results in characteristic epithelial blistering and have therefore become known as “*blebs*” proteins. Human Fraser syndrome presents with a similar phenotype and the *blebs* mice have been instrumental in identification of the genetic basis of Fraser syndrome. We have identified a new ENU-induced *blebs* allele resulting from a novel missense mutation in *Fras1*. The resulting mouse strain, *blood filled blisters* (*bfb*), presents with a classic *blebs* phenotype but does not exhibit embryonic lethality typical of other *blebs* mutants and in addition, we report novel palate and sternal defects. Analysis of the *bfb* phenotype confirms the presence of epithelial-mesenchymal adhesion defects but also supports the emerging role of *blebs* proteins in regulating signalling during organogenesis. The *bfb* strain provides new opportunities to investigate the role of Fras1 in development.

## Introduction

Fraser syndrome (OMIM 219000) is a rare autosomal recessive malformation spectrum defined by a combination of major features including cryptophthalmos, renal agenesis/malformation, genital anomalies and syndactyly although a range of additional features may also be present [1]. The growth in our understanding of both Fraser syndrome and mutant mice phenocopying Fraser syndrome features have been intimately linked and mutation in the *FRAS1* gene in human and mouse models of Fraser syndrome was simultaneously reported in both species [2], [3].

The Fraser syndrome mouse model was one of a series of mutant mice which have collectively become known as the “*blebs*” mutants. There are 4 classic *blebs* mutants that have been recognised, with each identifiable by variable levels of embryonic blistering, eye, renal and skeletal defects. The *blebs* mutant strain arose around 1970 in progeny of a mouse exposed to neutron radiation and named *blebbed* (*bl*). Over 30 years later the *bl* phenotype was demonstrated to be caused by a nonsense mutation in *Fras1*, which became the founding member of *blebs* proteins [2], [3]. Two additional blebs mutants arose spontaneously in colonies maintained at Jackson labs and were named *head blebs* (*heb*) and *eye blebs* (*eb*) with mutations in the genes *Frem1* and *Grip1*, respectively [4], [5], [6]. Finally, *myelencephalic blebs* (*my*), was identified in an X-ray-induced radiation mutant [7] and was subsequently shown to be caused by mutation in *Frem2* by genetic complementation [5].

Each of the *blebs* mutants have strongly overlapping phenotypes. Indeed, the *Fras1* and *Frem2* mutants have indistinguishable phenotypes and *Fras1*/*Frem2* double homozygous mice exhibit a spectrum of birth defects similar to those observed in single homozygotes, suggesting strongly overlapping functions [5]. Similarly, even though *heb* is the phenotypic outlier of the *blebs* group in that the phenotype is notably milder than the other *blebs* mice, the range of phenotypes observed in *heb* are completely encompassed by those exhibited by the other three *blebs* mutants [8]. These common phenotypes are indicative of a shared developmental function and each of the blebs mutant proteins have been demonstrated to participate in an epithelial/mesenchymal adhesive complex. Fras1, Frem2 and Grip1 are each expressed in epithelium while Frem1 and Frem3 are predominantly expressed in the mesenchyme. Each of these proteins has been shown to co-localise to the basement membrane [4], [9], [10].

Expression of each of the *blebs* proteins is required for the normal localisation of each family member to the basement membrane [11]. Loss of either Fras1, Frem1 or Frem2 results in delocalisation of all three proteins from the basement membrane and Fras1 and Frem1 each interact with Frem2 to form a complex [11]. Grip1 is structurally distinct from the Fras1/Frem family and is a cytoplasmic adaptor protein containing multiple PDZ domains which interact with the C-terminal cytoplasmic domains of Fras1 and Frem2 [4]. This interaction is necessary for the localisation of Fras1/Frem2 since Grip1 is required for the export of Fras1 to the basal surface of epidermal cells [4]. Thus, collectively, the Fras1, Frem and Grip1 proteins form an interdependent functional complex required for normal epithelial adhesion throughout the developing embryo.

The phenotypes observed in Fras1/Frem/Grip1 mutants clearly implicate these proteins in epithelial-mesenchymal adhesion and/or signalling. However, details of the role played by this complex in organogenesis remain elusive. For example, Fras1 is broadly expressed throughout the embryonic epithelium yet epithelial de-adhesion and blistering occurs at characteristic sites on the head and feet [2], [3]. This has been proposed to correlate with sites of friction within the uterine environment [8]. While this may well be the case, it is not a complete explanation since blistering is not commonly observed along the dorsal surface of the embryo which is in direct contact with the amnion throughout development. Further, while an overt blistering/de-adhesion phenotype is not seen within the embryonic kidney, renal agenesis and/or dysmorphology is a major component of the *blebs* and Fraser syndrome phenotypes. These observations suggest that the proposed signalling function of the Fras1/Frem/Grip1 complex may have an important role in organogenesis.

We performed an ENU mutagenesis screen in mice to identify genes important for organogenesis and which may therefore be candidates for causing human congenital defects [12]. This screen was designed to identify homozygous mutations that cause structural defects and mutant mice were harvested during embryonic development to guard against loss of mutants that may be embryonic lethal. We identified a new *blebs-like* strain which exhibits haemorrhagic blisters, cryptophthalmos and patterning defects in the feet which we have named *blood filled blisters* (*bfb*). Conventional linkage analysis followed by candidate gene sequencing identified a homozygous c10761T>C missense mutation resulting in an S3588P substitution within a domain of unknown function within Fras1. In addition to the classic *blebs* phenotypes, the *Fras1^bfb^* mutation results in a novel palatal defect and misalignment of the sternum. Existing *Fras1* mutant mice harbour null alleles and the *bfb* mutant mouse is therefore the first missense model of Fraser syndrome. The *bfb* strain will provide new opportunities to investigate the function of Fras1 in development and dysmorphology.

## Materials and Methods

### Mice

The details of *bfb* strain identification and gene mapping have been reported previously [12]. All mouse procedures were approved by the Royal Children's Hospital Animal Ethics Committee, RCH AEEC #A647 and #728.

### Genotyping

Adult ear clips and extraembryonic membranes were digested for genotyping in 0.4 mg/ml Proteinase K in Proteinase K buffer (10 mM Tris-HCl, pH7.5; 10 mM NaCl; 10 mM EDTA, pH8; 0.5% SDS) overnight at 55°C. DNA was then purified by isopropanol extraction for amplification and High Resolution Melt (HRM) analysis of *Fras1* exon 69 using primers *Fras1*.genF: 5′-TTTGACCTGCAGCTCTTGTG and *Fras1*.genR: 5′- GCTCGCCAGAGTTGATGAG. For HRM, a PCR reaction was performed in 10 µl containing 2.5 µl of 1/500 diluted gDNA, 5 µl Lightcycler®480 High Resolution Melting Master Mix (Roche), 3 mM MgCl_2_ and 0.2 µM of each primer. DNA was amplified for 45 cycles of 95°C for 10 sec, 62°C for 15 sec, 72°C for 5 sec. HRM melting curve data were obtained by slowly increasing the temperature from 75°C to 90°C at a rate of 0.02°C per second. Results were analysed by comparing each melt curve to control genotype DNA.

### *In silico* analysis of *Fras1* mutation

Pathogenicity of the *bfb* p.S3588P mutation was estimated using Polyphen (http://genetics.bwh.harvard.edu/pph/) where structural query options were set to default. A PSIC score of >2.0 indicates that that particular mutation was never or almost never observed in that protein family and would be classified as ‘probably-damaging’, scores of 1.5 to 2.0 classified as ‘possibly damaging’, and scores of <1.5 as ‘benign’. A second algorithm, SIFT (http://sift.jcvi.org/) was also used to predict the effect of the *bfb* p.S3588P amino acid substitution on protein function. A SIFT BLink analysis was performed using Fras1 protein ID NP_780682.3.

### Tissue Collection

Adult female mice were anaesthetised with isoflurane and culled by cervical dislocation according to the National Health and Medical Research Council Australian code of practice for the care and use of animals for scientific purposes (RCH AEEC approval #A647 and #A728). Embryos were dissected free from all extraembryonic membranes in Phosphate Buffered Saline (PBS), and processed depending on the subsequent application.

### Immunohistochemistry and immunoblotting

E15.5 *bfb* heads were processed by fixation in 4% paraformaldehyde (PFA) x 30 min and equilibrated in 30% sucrose/PBS solution for several hours/overnight with rotation until sunken. Tissues were then transferred to OCT Compound (Tissue-Tek), oriented appropriately and frozen in cold 2-methybultane (Merck). Sections of 10 µm were cut on a Leica CM3050-S cryostat (Leica microsystems), mounted on Superfrost Plus slides (Thermo Fisher Scientific) and stored at −80°C. Antigen retrieval was performed in 1% SDS, tissue was then permeabilised in 0.1% Triton X100, blocked in 0.1% Triton X-100/1% bovine serum albumin (BSA) in PBS, and incubated with a rabbit anti-Fras1 polyclonal antibody (1∶50; a kind gift from Georges Chalepakis) or rabbit IgG isotype control antibody (1∶250; Invitrogen) overnight at 4°C. Slides were then washed in PBS and incubated with Alexa Fluor 488-conjugated goat anti rabbit IgG (1∶1500; Molecular Probes). Following several washes in PBS, slides were mounted with Vectasheild (Vector Laboratories) and analysed using an Olympus IX70 fluorescent microscope (Olympus). Images were taken using an Evolution VF digital camera (MediaCybernetics).

Whole E13.5 wild-type, heterozygous and homozygous embryos were lysed by sonication (Vibra Cell, Sonics and Materials) in Laemlli SDS Sample Buffer plus 0.1 M DTT. A western blot was performed using standard procedures. Briefly, 100 µg of protein from each genotype was run through a 6 and 10% Tris-Glycine gel and transferred onto PDVF membrane (GE Healthcare) for 1 hour at 100 V. Membranes were preblocked with 5% non-fat milk and incubated with a rabbit anti-Fras1 polyclonal antibody (as above) or mouse anti-Human Transferrin Receptor monoclonal antibody (1∶1000; Invitrogen). Protein was detected with rabbit (1∶2000; Cell Signaling Technologies) or mouse (1∶10,000; Dako) HRP-linked secondary antibodies, respectively. Blots were processed using the Amersham ELC Prime detection kit (VWR International). Protein levels were calculated using ImageQuant TL, v7 software (GE Healthcare Life Sciences).

### Epifluorescence imaging of embryonic palates

Embryos were fixed in 4% PFA overnight, washed briefly in PBS and stained in a 100 µg/ml ethidium bromide solution for 30 sec. Tissue was briefly washed in fresh PBS before imaging using a Texas Red filter in a Leica M205FA fluorescence dissecting microscope. Images were converted to gray scale in Photoshop and gamma adjusted to optimise contrast.

### Hematoxylin and Eosin (H&E) Staining

E15.5 embryos were fixed in 4% PFA up to several weeks then transferred to 70% Ethanol prior to processing through xylene and paraffin using an Excelsior ES tissue processor (Thermo Scientific). Tissues were embedded in the required orientation using a Histocentre 2 Embedding Station (Shandon Life Sciences) and 8 µm sections cut on an MR-2 microtome (RMC products). A standard H&E protocol was followed with 5 min incubation in hematoxylin and 45 sec staining in eosin, and mounted with Entellan (Merck). Images were taken on a Nikon Eclipse 80 i microscope (Pathtech).

### *In situ* probe synthesis and hybridisation

Total RNA was isolated from adult mouse brain tissue using a Qiagen RNeasy Midi Kit (Qiagen). cDNA was generated from 1 µg RNA using random hexamers (Sigma) and BioScript™ reverse transcriptase (Bioline). cDNA fragments specific for the 3′UTR sequences of *Sox9* and *Tgfβ3* were amplified from RNA and ligated into pCR®II-TOPO using the TOPO® TA cloning kit (Invitrogen). An RNA probe was generated by plasmid amplification with M13 primers, synthesised with T7 or SP6 RNA polymerase using a MAXIscript® kit (Ambion), integrating dig-UTP (Roche). Probes were treated with Turbo DNase (Ambion) and unincorporated nucleotides removed using NucAway™ Spin Columns (Ambion). A sense probe was always generated to confirm specificity of the anti-sense probe at first-use.

E14.5 *bfb* heads were processed as for immunohistochemistry (above). *In situ* hybridisation on cryostat sections was performed as previously described [13], with modifications. Briefly, the *Tgfβ3 in situ* riboprobe was denatured at 70°C for 5 min prior to hybridisation, MABT was replaced with TBTX (50 mM Tris-Hcl; pH 7.5, 150 mM NaCl, 0.1% TritonX-100 in PBS), anti-digoxigenin-AP fragments antibody (Roche) was used at a dilution of 1∶1000 and tissue stained with nitroblue tetrazolium (NBT)/5-bromo-4-chloro-3-indolyl phosphate (BCIP). Once colour had developed slides were washed in PBS, rinsed briefly in 100% EtOH and mounted with Aquamount (Thermo Scientific). Images were taken using a Nikon Eclipse 80 i microscope (Pathtech).

E12.5 *bfb* embryos were processed for whole mount *in situ* hybridisation as previously described [14], with modifications. After methanol rehydration, embryos were permeabilised by incubation in 10 µg/ml proteinase K for 1 hr. Embryos were post-fixed in 0.2% glutaraldehyde/4% paraformaldehyde (PFA), equilibrated in prehybridisation buffer and incubated at 65°C overnight with *Sox9 in situ* riboprobe. After adequate washing to remove unbound probe, embryos were blocked with 10% sheep serum and incubated with an anti-digoxigenin-AP antibody (1∶1000, Roche). After additional washing, colour was developed with NBT/BCIP and the reaction stopped with EDTA buffer. The colour was fixed with 4% PFA, background staining reduced by storage in 100% methanol and images taken using a Nikon Eclipse 80 i microscope (Pathtech).

### Skeletal Preparations

Embryos >E16.5 were processed for skeletal preparations to visualise bone (Alizarin red) and cartilage (Alcian blue) as previously described [15], with modifications. All steps were carried out at room temperature. Embryos were skinned and eviscerated prior to fixation in 95% (v/v) ethanol. Tissues were incubated in Alcian blue stain (0.06% (w/v) Alcian blue in 80% (v/v) ethanol, 20% (v/v) acetic acid) overnight, re-fixed in 95% ethanol for several hours and cleared in 2% (w/v) KOH for 2 hr. Embryos were then incubated with Alizarin red solution (0.03% (w/v) Alizarin red in 1% (w/v) KOH) overnight then immersed in 1% (w/v) KOH/20% (v/v) glycerol solution for further clearing. For long-term storage, embryos were transferred into 50% (v/v) ethanol/50% (v/v) glycerol. Images were taken on a Leica MZ6 microscope using a Leica DFC290 camera (both Leica Microsystems Ltd).

A minimum of 3 independent embryos were processed for each analyses. Heterozygous embryos were indistinguishable to wild-type.

## Results

**Figure 1:**
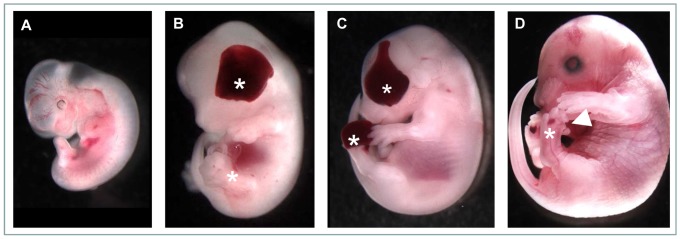
Representative *bfb* phenotypes at developmental age E11.5 (A), E13.5 (B), E15.5 (C) and E17.5 (D). Blood-filled blisters are evident across the eye and the distal hindlimbs (asterisks), although clear in the rear appendages at E13.5 and often distorting the digits in older embryos (arrowhead in D).

The ENU mutant strain reported here presented with clear fluid filled blisters over the lateral aspects of the head which are apparent around E12.5 and the most striking aspect of the phenotype which becomes apparent by E13.5 is the presence of blood filled blisters over the eyes (Figure 1B). At this time, clear fluid filled blisters also appear on the distal hind limbs. At E15.5 the head blisters are still prominent and the clear blisters present on the limbs have become hemorrhagic and are localised to the distal region of the hind feet and often involve the toes (Figure 1C). By E18.5 these blisters have typically resolved but residual pooling of blood can be detected under the skin, particularly over the feet (Figure 1D). Eyelid defects (cryptophthalmia [ie absence of palpebral fissure] or ablepharon) were observed in 74% of affected embryos older than E15.5. Hindlimb digit anomalies are also present, most commonly appearing as malpositioned digits but also manifest as preaxial polydactyly (see below). On the other hand, the forelimbs never present with hemorrhagic blisters or digit anomalies. The strongly penetrant hemorrhagic dermal lesions characteristic of the phenotype in this strain led to the name *blood filled blisters* (*bfb*).

**Figure 2:**
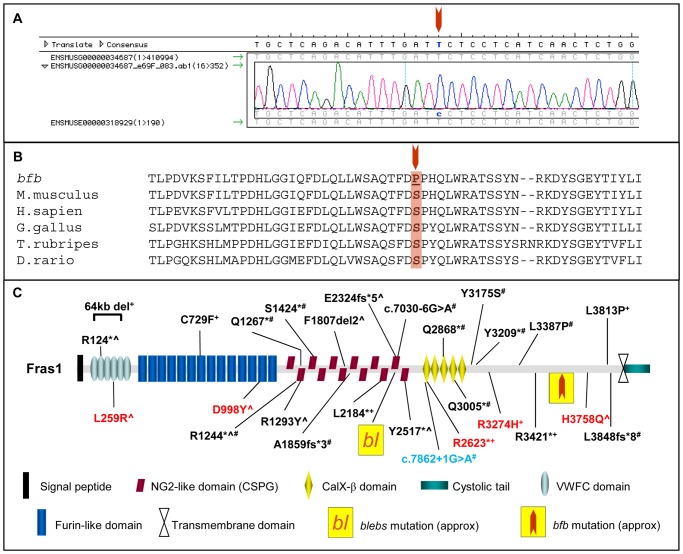
The *bfb* mouse harbours a mutation in Fras1. (A) Chromatogram of *Fras1* gene sequence identifying the c.10762T>C mutation in *Fras1^bfb/bfb^* mice. (B) Sequence alignment of the Fras1 protein sequence showing the amino acid residue affected in *Fras1^bfb/bfb^* is highly conserved across evolution. (C) Schematic of the Fras1 protein detailing protein domains, location of *Fras1^bfb/bfb^* mutation in the C-terminal region, the *blebs* mutation (*bl*), and reported human mutations (NM_025074). ^?^heterozygous,^+^compound heterozygous, ^#^homozygous, black; Fraser Syndrome, red; CAKUT, blue; Ablepharon macrostomia syndrome/Fraser Syndrome.

Mutation or deletion of either *Fras1*, *Frem 1, Frem2* or *Grip1* produces similar phenotypes to those observed in the *bfb* strain [8]. The high probability that *bfb* carries a mutation of one of these genes prompted a targeted SNP analysis resulting in strong linkage of the *bfb* phenotype to *Fras1* [12]. Sequencing of all 75 exons and flanking sequences from genomic DNA identified a c10762T>C mutation causing a Ser3588Pro substitution in *Fras1* (Figure 2A). Ser3588 is a highly conserved residue across diverse species ranging from fish to humans (Figure 2B). Prediction output from PolyPhen-2 for the *bfb* Fras1 p.S3588P mutation indicates a ‘probably damaging’ effect of the mutation on protein function, with a PSIC score of 0.999. SIFT prediction output denotes that *bfb* Fras1 p.S3588P amino acid change is ‘not tolerated’. A total of 520 embryos were examined for this study and the majority of 121 genotypically mutant embryos at E12.5 or older exhibited one or more mutant phenotypes while none of these phenotypes were observed in 249 heterozygous or 133 wildtype embryos (Table 1). In addition, homozygous wildtype, heterozygous and homozygous mutant embryos have been identified in expected ratios (25.6% *Fras1^+/+^*:47.9% *Fras1^+/bfb^*:26.5% *Fras1^bfb/bfb^*) indicating a negligible level of embryonic lethality.

**Table 1:** Phenotypes observed in *Fras1^bfb/bfb^* embryos.

Age	Ex	Head	Eyelid	Eye (R)	Eye (L)	FL	HL (R)	HL (L)	Pd	Ster	GCP	PB	Kid	Ph
**≤E11.5**	-	2/17 (12%)	-	-	-	-	-	-	1/17 (5%)	-	-	-	-	**2/17 (12%)**
**≥E12.5**	10/12 1 (8%)	17/121 (14%)	-	18/121 (15%)	25/121 (21%)	0/121 (0%)	30/121 (25%)	53/121 (44%)	16/121 (13%)	-	-	-	21/23 (91%)?	**96/121 (80%)**
**>E15.5**	-	-	28/38 (74%)	-	-	-	-	-	-	9/10 (90%)^#^	7/42 (17%)	19/23 (83%)*	-	**-**

Ex; exencephaly, FL; forelimbs, HL; hindlimbs, Pd; Polydactyly, Ster; sternum, GCP; gross cleft palate, PB; palate blister, Kid; no kidneys, Ph; any observed phenotype, R; right, L; left. ^#^≥E17.5, *Age E15.5 only, no palate blisters identified at E17.5 (4/4), ?includes 5/23 small/abnormal or only 1 kidney.

**Figure 3:**
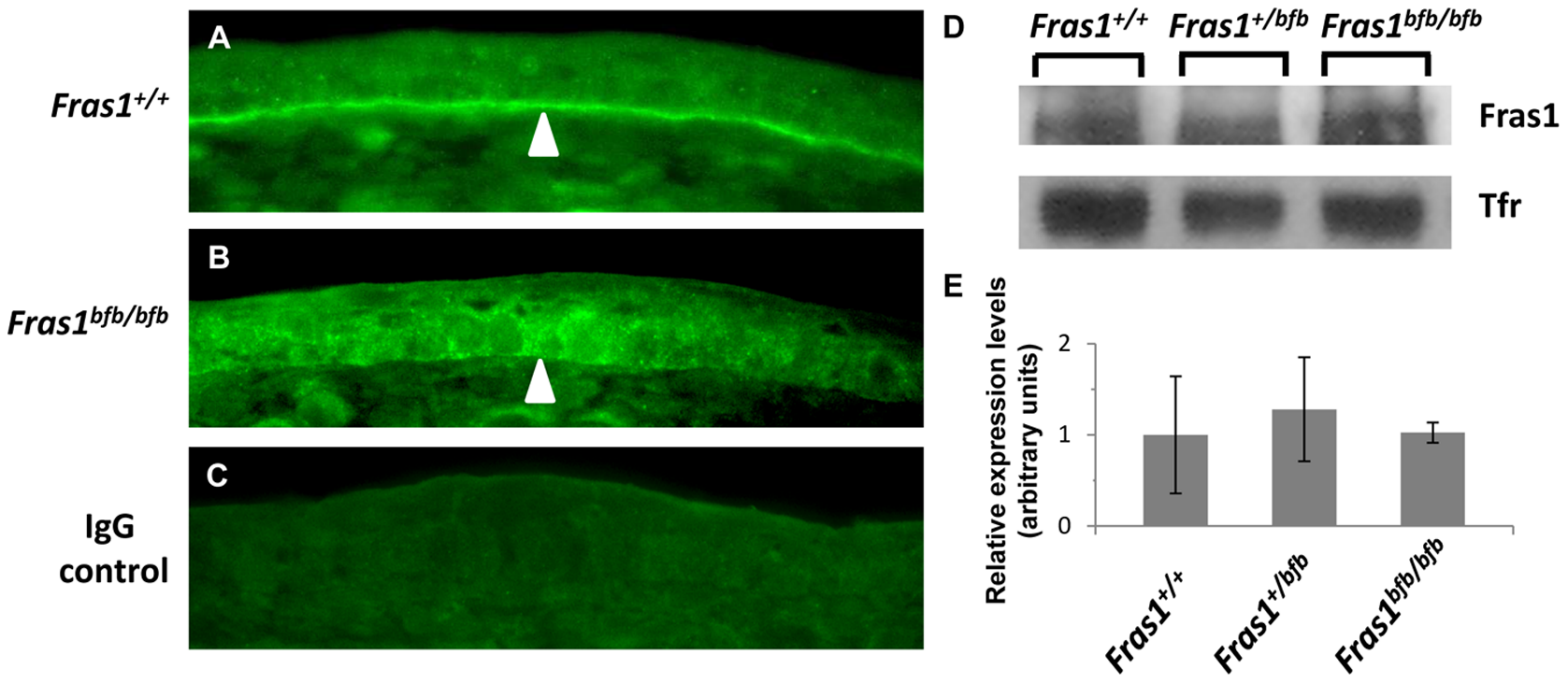
Immunofluoresence in E15.5 embryonic epidermis of the skin at the level of the kidneys. Fras1 expression in *Fras1^+/+^* (A) and *Fras1^bfb/bfb^* (B) shows mislocalisation of the protein to the basal membrane in mutant tissue (arrowheads). Rabbit IgG control (C). Western blot analysis indicates comparable levels of Fras1 protein in *Fras1^+/+^*, *Fras1^+/bfb^* and *Fras1^bfb/bfb^* samples (D and E).

The *Fras1* mutation localises to a C-terminal protein domain with no previously recognised structural motif or function (Figure 2C). Immunohistochemical analysis of *Fras1^+/+^* and *Fras1^bfb/bfb^* in embryonic epidermis demonstrates that Fras1 protein expression localises within the basal membrane of the skin in WT embryos. In contrast, Fras1 staining in *Fras1^bfb/bfb^* embryos is not well defined and appears mislocalised and diffuse in comparison to the WT staining (Figure 3A and B), indicating that the *Fras1* missense mutation disrupts the ability of Fras1 to correctly locate to the basal membrane. Levels of protein expression are well above background (Figure 3C) and western blot analysis indicates that although the Fras1 protein is not localised correctly in mutant embryos, expression levels are comparable in *Fras1^+/+^*, *Fras1^+/bfb^* and *Fras1^bfb/bfb^* samples (Figures 3D and E).

**Figure 4:**
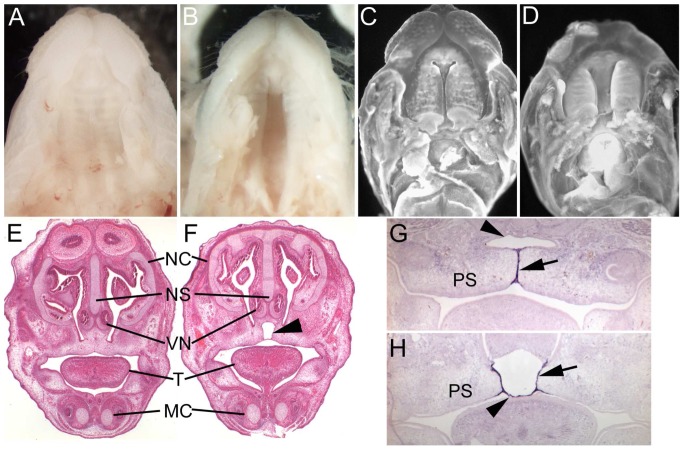
Morphology of the palate in *Fras1^+/+^* (A,C,E and G) and *Fras1^bfb/bfb^* (B,D,F and H) mice. At E18.5 the palate has fused completely in all wildtype foetuses (A) but a proportion of *Fras1^bfb/bfb^* mice have an overt palatal cleft (B). The palatal shelves have elevated and become closely opposed in wildtype mice (C) while there is a significant delay in palatal shelf growth in *Fras1^bfb/bfb^* embryos (D). At E15.5 there is a complete fusion between the palatal shelves and nasal septum in wildtype mice (E) while a subtle palatal blister is evident in 80% of E15.5 *Fras1^bfb/bfb^* embryos (arrowhead, F). *TGFβ3* is strongly expressed in palatal shelf epithelium (arrow) of both wildtype (G) and *Fras1^bfb/bfb^* mutants (H) but is aberrantly expressed in the nasal septum epithelium (arrowhead) in mutants. NC, nasal cavity; NS, nasal septum; VN, vomeronasal organ; T, tongue; MC, Meckel's cartilage, PS, palatal shelf.

Examination of E18.5 embryos revealed that 17% of mutant embryos have an overt cleft secondary palate (Figure 4A and B). The majority of these exhibit a complete cleft but in a small proportion of cases a partial cleft is apparent resulting in an anterior fistula (not shown). Formation of a continuous palate begins at approximately E13.5 when the individual palatal shelves elevate from their position adjacent the tongue to become opposed. By E14.5 continued growth of the shelves results in a close approximation of opposing epithelial surfaces as a prelude to fusion (Figure 4C). However, in *Fras1^bfb/bfb^* mutant embryos, growth of the palatal shelves appears delayed resulting in a significantly larger gap between opposing palatal epithelia compared to controls (Figure 4D). Histological analysis of E15.5 palates, well after palatal fusion, reveals complete removal of the epithelial seam and complete fusion between the nasal septum and palatal shelves in wildtype embryos (Figure 4E). However, in *Fras1^bfb/bfb^* embryos without an overt cleft palate, 83% of E15.5 individuals examined (n = 23) exhibited an abnormal fusion outcome resulting in formation of a blister-like structure between the palatal shelves and nasal septum (Figure 4F, Table 1). The extent of the blister was variable but was always restricted to the anterior portion of the palate associated with the nasal septum. These blisters were never observed in wildtype or heterozygous littermates. *Tgfβ3* is specifically expressed in the medial edge epithelium between opposing palatal shelves and is required for activation of the epithelial adhesion mechanism responsible for initial fusion of palatal shelves [16], [17], [18], [19]. We therefore examined *Tgfβ3* to determine if this clefting phenotype resulted from alteration in *Tgfβ3* expression. *Tgfβ3* is robustly expressed in the medial edge epithelium of both *Fras1^bfb/bfb^* and wildtype littermates at E14.5 (Figure 4G and H). However, in *Fras1^bfb/bfb^* embryos, while the palatal shelf epithelium is closely associated with the underlying mesenchyme, the epithelium normally associated with the nasal septum epithelium has completely delaminated. In addition, the mutant nasal septum epithelium strongly expresses *Tgfβ3* while in the wildtype epithelium, which is still in close association with the underlying mesenchyme, no *Tgfβ3* expression is observed. Thus, *Fras1^bfb/bfb^* embryos exhibit a palatal defect which ranges from an isolated sub-epithelial blister to complete cleft secondary palate. Cleft of the lip and/or primary palate was not observed in any *Fras1^bfb/bfb^* embryos.

**Figure 5:**
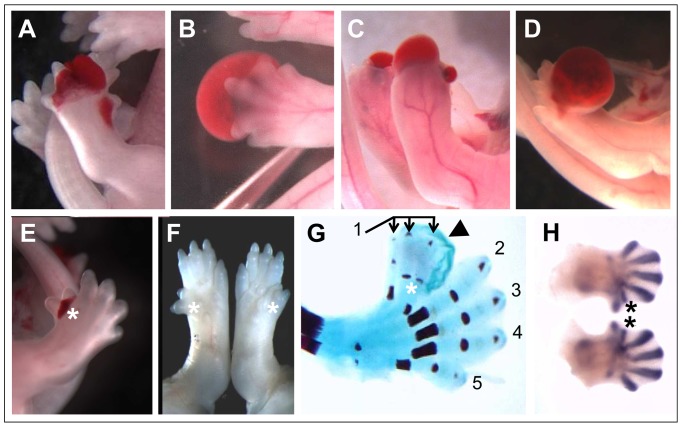
Preaxial polydactyly and blood-filled blisters in the hindlimbs of *Fras1^bfb/bfb^* mice. Blood-filled blisters are evident in the hindlimbs of *Fras1^bfb/bfb^* at E14.5 (A and B) and E17.5 (C and D), often distorting the digits. A proportion of *Fras1^bfb/bfb^* exhibit preaxial polydactyly (asterisks in E-H), evident in E14.5 and E17.5 wholemounts (E and F), E17.5 skeletal preparations (G) and E12.5 Sox9 in situ stained embryos (H). Evidence of a blister can also be seen in an E17.5 skeletal preparation (arrowhead in G). Digit numbers are indicated in (G).

Extensive blistering was observed on the distal hindlimb autopod in a high proportion of >E12.5 *Fras1^bfb/bfb^* embryos (Table 1). The blisters occurred either on the dorsal or ventral surface of the foot and were commonly associated with malpositioned digits (Figure 5A and B). Blisters were also observed over the distal tip of the foot completely enclosing the digits (Figure 5C). The foot blisters typically resolved but in some cases substantial blisters were still present as late as E17.5 (Figure 5D). Blisters were never seen on the forelimbs. Similarly, while 13% of *Fras1^bfb/bfb^* embryos exhibited overt preaxial polydactyly on the hindlimbs, polydactyly was never observed in forelimbs (Figure 5E and F). Typically this involved a single additional digit adjacent to digit I but in rare cases more than one accessory digit was observed (Figure 5G). The accessory digit was often associated with a blister in the same general region of the autopod (Figure 5E and G). Delamination of the skin in the hindlimbs is first observed at E12.5 and these blisters are filled with clear fluid. Interestingly, visualisation of chondrogenic condensations at E12.5 reveals that accessory digits are already specified at this stage although they are not yet apparent by gross examination (Figure 5H).

**Figure 6:**
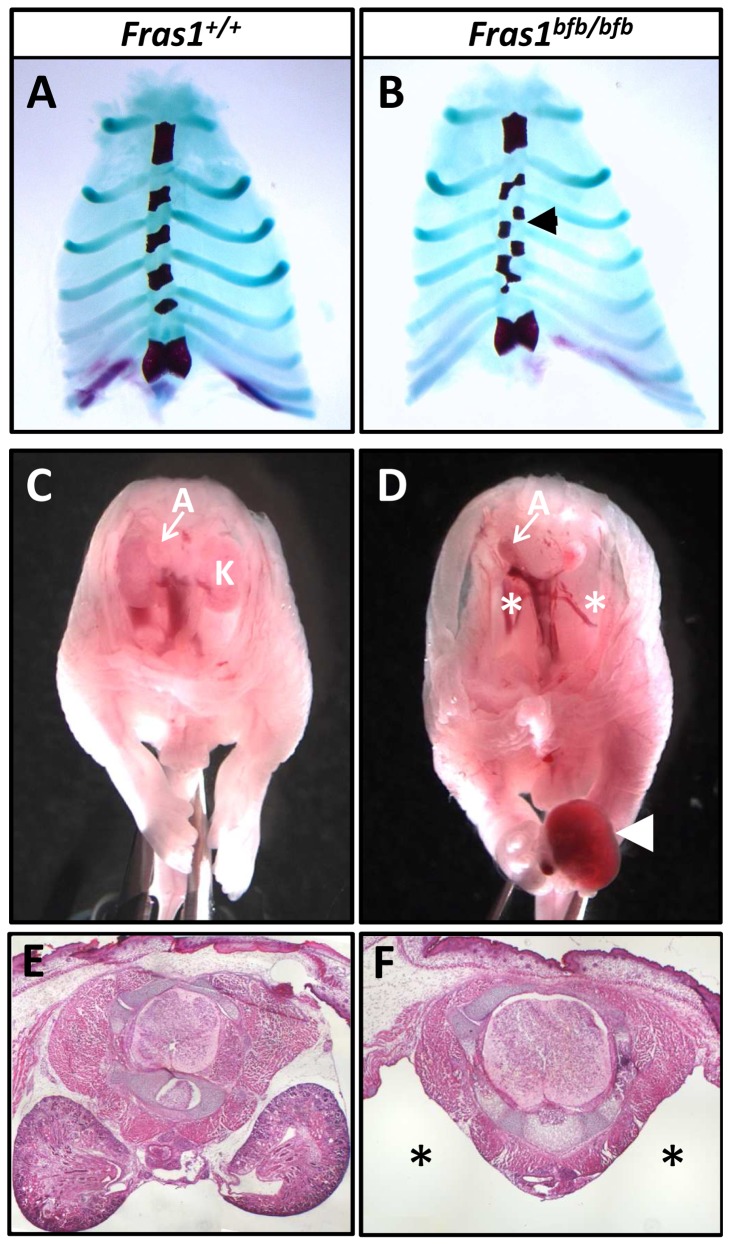
Sternum and kidneys are abnormal in *Fras1^bfb/bfb^* embryos. Sternabrae are misaligned in *Fras1^bfb/bfb^* mice at E18.5 (B), when compared to the sternum of *Fras1^+/+^* embryos of the same age (A). Analysis of the internal organs of *Fras1^+/+^* (C and E) and *Fras1^bfb/bfb^* (D and F) mice show no identifiable kidneys in the majority *Fras1^bfb/bfb^* mutants (asterisk in D). A blood-filled blister is also present in the hindlimb of this embryo (arrowhead in D). Transverse sections of an E17.5 *Fras1^bfb/bfb^* embryo show a large void where the kidney should be located (asterisk in F). A, adrenal gland; K, kidneys.

Skeletal preparation of *Fras1^bfb/bfb^* embryos did not reveal any gross skeletal malformation other than that observed in the hindlimb feet. However, while the rib cage was normal in appearance and was made up of the appropriate number of ribs, the sternum in 90% of *Fras1^bfb/bfb^* mice examined (n = 10) showed a subtle structural anomaly. The sternum forms from the fusion of two independently developing anlagen which arise in isolation from the rib primordia. These primordia then come together at the ventral midline to fuse in a cranial to caudal manner [20]. In all *Fras1^bfb/bfb^* embryos examined, the 2^nd^-5^th^ sternebrae were bipartite and the corresponding ribs misaligned (Figure 6A and B). The 1^st^ (manubrium) and last (xiphoid) sternebrae appeared normal. The costavertebral articulations were also normal.

Examination of wholemount *Fras1^bfb/bfb^* embryos revealed absent or severely reduced kidneys in 91% of mutant samples (Figure 6C and D; Table 1). Histological examination confirmed that there was no residual kidney material in the majority of cases (Figure 6E and F) although a small mass of kidney material was identified in 5/21 mutant embryos.

## Discussion

ENU mutagenesis is a powerful, phenotype-driven screening strategy that can be tailored to individual research questions by focussing only on phenotypes that involve the system under examination. We carried out an embryonic lethal screen to identify recessive mutations causing birth defects and identified a strain with a classic *blebs* phenotype and mutation in the Fraser syndrome gene *Fras1*. Disease causing mutations in *FRAS1* do not appear to be clustered within any particular region of the gene and can be found scattered throughout the open reading frame, although more than half of these are nonsense mutations (Figure 2C) [21], [22], [23], [24], [25], [26], [27]. The Ser3588Pro substitution in *Fras1* that resulted in the *bfb* strain resides within the C-terminus in a structurally unremarkable domain. Some of the previously described disease causing missense mutations also localise to this region of FRAS1 indicating that the lack of identifiable domain structure belies the functional importance of this intervening structure which links the transmembrane domain region to the structurally better characterised N-terminus. Indeed, the delocalisation of Fras1 from the basement membrane suggests this region of Fras1, while unlikely to be directly involved in interactions with the Frem proteins, may never the less be important in maintaining Fras1 in the correct configuration to participate in these interactions. Further, this data suggests that the mechanism of pathogenesis in *bfb* involves disruption of the entire blebs complex since all of the components are co-dependent on each other for normal localisation [11].

The *bfb* strain was initially identified because of the presence of cleft secondary palate. An overt cleft secondary palate is observed in around 17% of *Fras1^bfb/bf^* mutants while 80% exhibit abnormal fusion histologically. The compromised epithelial-mesenchymal integrity characteristic of all *blebs* mutants is likely to underlie this phenotype. Tgfβ3 is normally expressed in both the palatal shelf and nasal septum epithelia beginning before palatal fusion (prior to E13.5) and is still readily detectable in the palatal shelves at E14.5, but downregulated in the nasal septum epithelium at this age [28]. Consistent with this we do not see *Tgfβ3* in the nasal septum epithelium of wildtype control mice at E14.5. However, *Tgfβ3* is robustly expressed in the delaminated nasal septum epithelium of *Fras1^bfb/bfb^* embryos. These *Fras1^bfb/bfb^* embryos do not appear to exhibit any global developmental delay suggesting that the persistence of *Tgfβ3* expression in the nasal septum epithelium may not simply be a delay of the normal downregulation. It is possible that the delamination of the nasal septum epithelium disrupts some form of epithelial-mesenchymal interaction that normally extinguishes *Tgfβ3* expression in this region. Interestingly, the nasal septum and palatal shelf epithelia have fused in the mutants. This suggests that the initial fusion event occurred normally but that the nasal septum epithelium secondarily delaminated from the underlying mesenchyme, possibly due to the increased physical forces associated with fusion. The reduced growth of pre-fusion palatal shelves in mutant embryos may contribute to this phenomenon since a delay in convergence at the midline is likely to alter the normal dynamics of fusion. The origins of the reduced growth in the palatal shelves are unclear since histological examination has not revealed any clear evidence of gross epithelial delamination. However, growth factor signalling between the epithelial and mesenchymal components of the palatal shelves have been demonstrated to have essential roles in proliferation [29], [30]. It is therefore possible that the compromised epithelial-mesenchymal interaction at the surface of the nascent *Fras1^bfb/bfb^* palatal shelves alters one or more signalling events that are required for normal growth thereby contributing to the failure of normal palate fusion.

Syndactyly is a feature of both Fraser syndrome and *blebs* mutants. However, we do not observe clearly evident syndactyly in *bfb* mutants. Our analysis has been restricted to embryonic ages only and embryos such as that depicted in Figure 4C may go on to develop syndactyly. The *Fras1^bfb/bfb^* mutation does however result in hindlimb preaxial polydactyly in 13% of mutant embryos. The presence of characteristic hemorrhagic blisters on the hindlimbs appears to be a strong argument for a causative relationship between blisters and digit anomalies. We found that twice as many embryos exhibit foot blisters as have polydactyly. This may simply be a reflection of the variation in extent or location of the blister and its ability to disrupt normal digit patterning. However, there appears to be a strong bias towards occurrence of blisters on the left hindlimb (44%) versus the right (25%). If the existence of blisters were the cause of the polydactyly, the ratio of affected polydactylous feet would be expected to follow the same trend. In fact we observe a strong bias towards right sided polydactyly (13/16). The significance of these biases is unclear but they do raise the possibility that the origins of the polydactyly may be more complex than simple disruption of the limb bud epithelium. We observe polydactyly as early as E12.5 in *Sox9*-positive chondrogenic condensations and have identified expanded hindlimb buds in mutants at E11.5 in a small number of cases. Digit specification occurs between E10.5 and E11.5 [31] and the earliest observable blisters on hindlimbs appear at E12.5. It is therefore possible that the polydactyly and blister phenotypes are not directly linked and that polydactyly occurs from a separate signalling defect that impacts on limb bud patterning around E10.5. The role of *blebs* proteins in growth factor signalling is still emerging and is best illustrated by the recently described interaction between Frem1 and Gdnf signalling during kidney maturation [32]. Recent data has demonstrated that Frem1, and by implication the other *blebs* proteins within the complex, are involved in regulation of *Gdnf* expression via interaction between the basement membrane proteins, nephronectin and integrin α8β1 [32]. Gdnf and integrin α8β1 are both essential for normal kidney development [33], [34] and these findings provide a mechanism by which all Fraser syndrome/*blebs* mutations could cause kidney agenesis/dysgenesis through altered signalling. Preaxial polydactyly results from ectopic Shh signalling in the anterior limb bud [35], [36]. The anterior of the limb bud is normally kept Shh-free through induction of mesenchymal factors such as Twist1, the expression of which is induced or sustained by ectodermal growth factor signals [37], [38], [39]. Thus, disruption of this epithelial-mesenchymal interaction could result in preaxial polydactyly independently of overt blistering as a result of disrupted signalling.

Detailed analysis of *Fras1^bfb/bfb^* skeletons does not reveal any overt skeletal dysplasia, similar to previously characterised *Fras1* mutants. However, 90% of *bfb* mutants exhibit a defect of the sternum that results in misalignment of the ribs. Overall the thoracic cage is normal in appearance indicating that the ribs themselves are unaffected. The sternum forms from two independent anlage which initially contact at the cranial end and fusion progresses in a cranial to caudal direction [40]. While the sternal anlagen and ribs arise independently, at the time of fusion the rib primordia have already become associated with the left and right sternal primordia. The sternum subsequently becomes segmented through the influence of the inserted rib primordia, with each rib inhibiting chondrocyte hypertrophy in the sternum opposite its point of contact [41]. This suggests that misalignment of the ribs and hemi-sternebrae seen in *Fras1^bfb/bfb^* embryos occurs as a result of abnormal progression of sternal fusion rather than inappropriate insertion of rib primordia. Sternal fusion begins at the rostral end with formation of the manubrium and ends with formation of the xiphoid process. Both the manubrium and xiphoid appear normal in *Fras1^bfb/bfb^* embryos and it is only the 4 intervening sternebrae that appear to be affected. Sternal clefts are a relatively common congenital defect in humans resulting from a failure of the fusion process. No *Fras1^bfb/bfb^* embryos exhibited a cleft sternum indicating that the fusion process itself is not compromised. Little is known about the mechanism regulating sternal fusion or the correct alignment of left and right ribs with each other. The observation of this phenomenon in *bfb* mutant embryos suggests that Fras1 plays a role in this process despite the absence of any known epithelial-mesenchymal interaction in the sternal fusion process.

The *bfb* mouse is the first ENU-induced *Fras1* allele and the first missense mutation in a mouse model of *Fras1*-mediated Fraser syndrome. The recovery of the expected proportion of homozygous mutant *bfb* embryos indicates a negligible level of embryonic death in contrast to that previously reported in other *Fras1* mutants [2], [3], [42] suggesting that the *bfb* missense mutation may have a somewhat less deleterious impact than the previous null alleles. Our analysis of *bfb* mutants has also revealed a number of additional phenotypes not previously described. The *bfb* strain provides a useful alternative model for Fraser syndrome and for the study of the role played by *blebs* proteins during embryonic development.
